# Supplementary material for: Are E-cigarettes associated with postpartum return to smoking? Secondary analyses of a UK pregnancy longitudinal cohort
Source: BMJ Open. 2022 Apr 11;12(4):e061028. doi: 10.1136/bmjopen-2022-061028 (PMC9006833; doi:10.1136/bmjopen-2022-061028)

Figure S1 – Consort diagram NB: five self-reported non-smokers from FU1 for PLS National were removed from n=167 as they quit more than 3 months before pregnancy

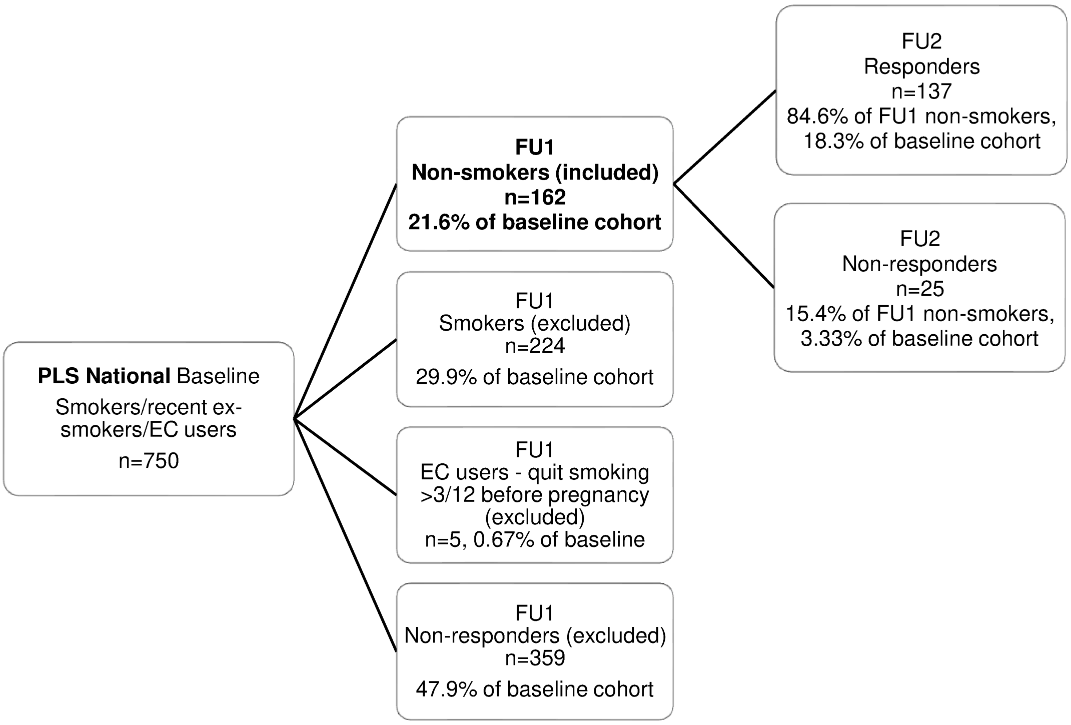

Supplement: Supplementary data [file bmjopen-2022-061028supp001.pdf]
