# Supplementary material for: Are E-cigarettes associated with postpartum return to smoking? Secondary analyses of a UK pregnancy longitudinal cohort
Source: BMJ Open. 2022 Apr 11;12(4):e061028. doi: 10.1136/bmjopen-2022-061028 (PMC9006833; doi:10.1136/bmjopen-2022-061028)
Supplement: Author's manuscript [file bmjopen-2022-061028.draft_revisions.pdf]

BMJ Open is committed to open peer review. As part of this commitment we make the peer review history of every article we publish publicly available.

When an article is published we post the peer reviewers' comments and the authors' responses online. We also post the versions of the paper that were used during peer review. These are the versions that the peer review comments apply to.

The versions of the paper that follow are the versions that were submitted during the peer review process. They are not the versions of record or the final published versions. They should not be cited or distributed as the published version of this manuscript.

BMJ Open is an open access journal and the full, final, typeset and author-corrected version of record of the manuscript is available on our site with no access controls, subscription charges or pay-per-view fees (http://bmjopen.bmj.com).

If you have any questions on BMJ Open's open peer review process please email info.bmjopen@bmj.com

BMJ Open

E-cigarette use and its association with postpartum return to smoking in a UK cohort

Journal:	*BMJ Open*
Manuscript ID	bmjopen-2022-061028
Article Type:	Original research
Date Submitted by the Author:	18-Jan-2022
Complete List of Authors:	Orton, Sophie; University of Nottingham<br>Taylor, Lauren; University of Nottingham, School of Medicine<br>Laing, Libby; University of Nottingham, School of Medicine<br>Lewis, Sarah; University of Nottingham, School of Medicine<br>Ussher, Michael; St George's University of London, Division of Population Health Sciences and Education; University of Stirling Institute for Social Marketing and Health<br>Coleman, Tim; University of Nottingham, School of Medicine<br>Cooper, Sue; University of Nottingham, School of Medicine
Keywords:	PRIMARY CARE, PREVENTIVE MEDICINE, PUBLIC HEALTH, REPRODUCTIVE MEDICINE

SCHOLARONE™
Manuscripts

I, the Submitting Author has the right to grant and does grant on behalf of all authors of the Work (as defined in the below author licence), an exclusive licence and/or a non-exclusive licence for contributions from authors who are: i) UK Crown employees; ii) where BMJ has agreed a CC-BY licence shall apply, and/or iii) in accordance with the terms applicable for US Federal Government officers or employees acting as part of their official duties; on a worldwide, perpetual, irrevocable, royalty-free basis to BMJ Publishing Group Ltd ("BMJ") its licensees and where the relevant Journal is co-owned by BMJ to the co-owners of the Journal, to publish the Work in this journal and any other BMJ products and to exploit all rights, as set out in our licence.

The Submitting Author accepts and understands that any supply made under these terms is made by BMJ to the Submitting Author unless you are acting as an employee on behalf of your employer or a postgraduate student of an affiliated institution which is paying any applicable article publishing charge ("APC") for Open Access articles. Where the Submitting Author wishes to make the Work available on an Open Access basis (and intends to pay the relevant APC), the terms of reuse of such Open Access shall be governed by a Creative Commons licence – details of these licences and which Creative Commons licence will apply to this Work are set out in our licence referred to above.

Other than as permitted in any relevant BMJ Author's Self Archiving Policies, I confirm this Work has not been accepted for publication elsewhere, is not being considered for publication elsewhere and does not duplicate material already published. I confirm all authors consent to publication of this Work and authorise the granting of this licence.

For peer review only

E-CIGARETTE USE AND ITS ASSOCIATION WITH POSTPARTUM RETURN TO SMOKING IN A UK COHORT

Sophie Orton PhD[1]*, Lauren Taylor BMBS[1], Libby Laing PhD[1], Sarah Lewis Prof[1], Michael Ussher Prof[2, 3], Tim Coleman Prof[1], Sue Cooper PhD[1]

1. School of Medicine, University of Nottingham, Nottingham, UK

2. Population Health Research Institute, St George's, University of London, London SW17 0RE, UK.

3. Institute for Social Marketing and Health, University of Stirling, Stirling FK9 4LA, UK.

* Corresponding author: Dr Sophie Orton, University of Nottingham, Sophie.orton@nottingham.ac.uk

Word count: 1907

For peer review only

ABSTRACT

Objectives: Postpartum return to smoking (PPRS) is an important public health problem. E-cigarette (EC) use has increased in recent years, and in a contemporary UK pregnancy cohort, we investigated factors, including ECs use, associated with PPRS.

Design: Secondary analyses of a longitudinal cohort survey with questionnaires at baseline (8–26 weeks' gestation), late pregnancy (34–36 weeks) and 3 months after delivery.

Setting: 17 hospitals in England and Scotland in 2017.

Participants: The cohort recruited 750 women who were current or recent ex-smokers and/or EC users. A subgroup of women reported being abstinent from smoking in late pregnancy (n=162, 21.6%), and of these 137 (84.6%) completed the postpartum questionnaire and were included in analyses.

Outcome measures: Demographics, smoking behaviours and beliefs, views and experience of ECs and infant feeding.

Results: 35.8% (95% CI 28-44%) of women reported PPRS. EC use in pregnancy (adjusted OR 0.34, 95% CI 0.13-0.85) and breastfeeding (adjusted OR 0.06, 95% CI 0.02-0.24) were inversely associated with PPRS, whilst household member smoking at 3 months postpartum was positively associated with PPRS (adjusted OR 11.1, 95% CI 2.47-50.2).

Conclusion: EC use in pregnancy could influence postpartum return to smoking. Further research is needed to confirm this and investigate whether ECs could be used to prevent PPRS.

For peer review only - http://bmjopen.bmj.com/site/about/guidelines.xhtml

Strengths and limitations of this study

- This is the first study, to our knowledge, to explore use of ECs in pregnancy and postpartum, among other potential risk factors, for return to smoking postpartum.
- The cohort collected longitudinal, prospective data on women's smoking behaviour during pregnancy and postpartum, reducing recall error.
- The cohort is likely to be broadly representative as the demographic profile of smokers is similar to other UK pregnancy cohorts.
- The primary study was not designed to answer the research question; consequently, relatively small numbers of women reported returning to smoking postpartum and using ECs.
- Smoking and EC use were self-reported, which could lead to underreporting due to social stigma; however the surveys were completed anonymously which may have minimised this issue.

For peer review only

For peer review only - http://bmjopen.bmj.com/site/about/guidelines.xhtml

INTRODUCTION

Helping pregnant women quit smoking and remain abstinent in the long term is an important public health issue. In 2019/2020 12.1% of women in England were smoking in early pregnancy[1], however around half attempt cessation after conception[2]. Unfortunately, relapse is common, with up to 75% returning to smoking within 12 months of giving birth[3-5]. Reducing maternal postpartum return to smoking (PPRS) would have substantial health benefits for both mother and baby[6]; women would reduce their own risks of smoking related illness, reduce ill health and mortality associated with second hand smoke exposure among their children[7 8], and reduce the likelihood of their children becoming smokers themselves in later life[9].

There are currently no effective interventions for reducing PPRS[10]. Understanding the factors that are associated with PPRS is essential for the development of targeted and evidence-based interventions. A systematic review[11] observed that women that returned to smoking postpartum tended to be less well educated, younger, multiparous, living with a partner or household member who smoked, experiencing higher stress, depression or anxiety, not breastfeeding, intending to quit only for pregnancy, and having low confidence in remaining quit after giving birth.

Since this review, e-cigarettes (ECs) have become increasingly popular [12]. ECs have potential for public health benefit as they do not involve tobacco combustion, which is the main source of harm from conventional cigarettes. In non-pregnant smokers, there is moderate certainty that ECs containing nicotine improve cessation rates, compared with non-nicotine vaping products or nicotine replacement therapy (NRT)[13]. In 2021, ECs were used in 29% of UK quit attempts[12] and around 5% of UK women report using ECs in pregnancy[14]. There is also evidence from qualitative studies that some women are using ECs to avoid returning to smoking after having their baby[15]. Hence, using a contemporary UK pregnancy cohort, we investigated factors associated with PPRS, including EC use in pregnancy and afterwards[11].

For peer review only - http://bmjopen.bmj.com/site/about/guidelines.xhtml

METHODS

This paper follows the STROBE reporting guidelines[16] (S1). We conducted secondary analyses on data collected in a UK longitudinal pregnancy cohort designed to explore the use of and attitudes towards ECs during pregnancy and postpartum. Full methods and cohort characteristics are presented elsewhere[14]. In brief, the cohort recruited women from 17 hospital antenatal clinics from June to November 2017. Hospitals had varying smoking in pregnancy rates and were from a range of geographical locations in England and Scotland. Screening surveys were systematically handed out to all pregnant women attending selected antenatal clinics. Women were eligible to join the cohort if they self-reported being either current or recent ex-smokers (quit smoking in the 3 months prior to pregnancy), and/or current EC users. Data were collected at 8-26 weeks gestation (baseline), 34–36 weeks gestation (follow-up one, FU1) and 12 weeks postpartum (follow-up two, FU2). Baseline questionnaires asked about maternal demographics (age, education, ethnicity), parity, smoking behaviours and beliefs, and use of ECs. FU1 and FU2 questionnaires were similar to baseline but also asked about plans for infant feeding, how baby was being fed and smoking/EC use postpartum.

Patient and Public Involvement

Patient and public involvement colleagues contributed to the design of the study, including providing feedback on the importance of the research question, designing study materials and advising on study recruitment.

Ethics approval

Ethical approval was granted by the South West Frenchay Research Ethics Committee (IRAS project ID 222886).

Selection of respondents for inclusion in analyses

Women who reported having quit smoking either in the 3 months prior to pregnancy, or during pregnancy, and were still abstinent by FU1 (34-36 weeks gestation) were included in the analysis.

For peer review only - http://bmjopen.bmj.com/site/about/guidelines.xhtml

Dependant and explanatory variables

The primary outcome measure was return to smoking at 3 months postpartum using participant's responses to the question 'which statement best describes your smoking right now?'. Response options were 'I do not smoke at all' 'I smoke occasionally, but not every day', 'I smoke every day, but less than when I was pregnant', 'I smoke every day, about the same as when I was pregnant' and 'I smoke every day and tend to smoke more than when I was pregnant'. Women who reported smoking at least occasionally were considered to have returned to smoking.

The explanatory variables measured at baseline/during pregnancy were: baseline smoking status (current smoker or recent ex-smoker), education ('no qualifications' or 'General Certificate of Secondary Education or above', using highest educational qualification), maternal age, previous pregnancies (no previous pregnancy' or 'previous pregnancy'), baseline Heaviness of Smoking Index (HSI) ('low', 'moderate' or 'high')[17], NRT use in pregnancy ('not used NRT' or 'used NRT'), confidence in staying smoke free ('not at all – a little' or 'moderately – extremely'), whether they had accessed stop smoking support during pregnancy ('not accessed'' or 'accessed'), and EC use during pregnancy ('never used EC in pregnancy' or 'used EC at some point during pregnancy'). Postpartum explanatory variables were breastfeeding ('baby no longer/never breastfed' or 'baby still breastfeeding at 12 weeks postpartum'), household member smoking ('no household member smoking' or 'household member smokes' and EC use ('never used EC postpartum' or 'used EC at some point postpartum').

Analysis

Analyses were conducted using Stata version 13.1[18]. We handled missing data using available case analysis. Baseline demographic characteristics were analysed using descriptive statistics. Prevalence of PPRS was calculated for those who responded at FU2.

For peer review only - http://bmjopen.bmj.com/site/about/guidelines.xhtml

An exploratory logistic regression was conducted to identify factors associated with PPRS. Collinearity between variables was assessed using correlation analysis and no significant correlations were identified. HSI was excluded from logistic regression because all those in the PPRS group had low HSI. We used an iterative approach to determine which variables best predicted PRRS, first determining which variables were significantly associated ($p < 0.05$) with PPRS in univariate logistic regression. These variables were then included together in a multivariate logistic regression model. Variables that were not significant were removed from the model, so that only significant variables ($p < 0.05$) were retained. Variables which had been excluded, including those that were not significant in univariate analysis, were added into the model consecutively to assess whether they became significant and retained if so. Effects was expressed as odds ratios and 95% confidence intervals.

RESULTS

Response rates

The total cohort included 750 women, and 162 (21.6%) were eligible and included in analyses. From these eligible women, response rate for the FU2 (postpartum) survey was 84.6% (137/162) (Figure S1).

Cohort characteristics

Table 1 presents the baseline characteristics of women who were abstinent from smoking in late pregnancy (i.e., at FU1) and of respondents and non-respondents to the postpartum questionnaire (FU2). At baseline, 17.9% smoked, 82.4% had low addiction levels and 93.8% were not using ECs. 29.6% used an EC at some point in pregnancy, and 19.9% used an EC at some point in the 12 weeks since having their baby. Those who did not respond to FU2 were more likely to be a current smoker at baseline and have a lower educational level.

Table 1: Baseline questionnaire characteristics of those participants who were non-smokers in late pregnancy and a comparison between those who did and did not respond to the postpartum survey

**Characteristics at baseline (8-26 weeks gestation)**	**Women abstinent from**	**Responded to postpartum questionnaire**	**Did not respond to**

For peer review only

	smoking in late pregnancy N (%) N= 162	N (%) N=137	postpartum questionnaire N (%) N=25
**Smoking status**			
Recent ex-smoker	133 (82.1)	116 (84.7)	17 (68.0)
Current smoker	29 (17.9)	21 (15.3)	8 (32.0)*
**HSI score**			
Low addiction	28 (82.4)	22 (84.6)	6 (75.0)
Moderate addiction	6 (17.7)	4 (15.4)	2 (25.0)
High addiction	0 (0.0)	0 (0.0)	0 (0.0)
**Education**			
No qualifications	11 (6.8)	7 (5.15)	4 (16.0)
GCSE or above (including other)	150 (93.2)	129 (94.9)	21 (84.0)*
**Maternal age (Mean, SD)**	26.9 (5.6)	27.1 (5.3)	25.9 (7.0)
**Ethnicity**			
White British	139 (86.3)	118 (86.8)	21 (84.0)
Other ethnicity	22 (13.7)	18 (13.2)	4 (16.0)
**Previous pregnancy**			
No previous pregnancy	73 (45.6)	64 (47.1)	9 (37.5)
Previous pregnancy	87 (54.4)	72 (52.9)	15 (62.5)
**Confidence in staying smoke free**			
Not at all-a little	20 (12.7)	18 (13.5)	2 (8.33)
Moderately-extremely	137 (87.3)	115 (86.5)	22 (91.7)
**EC use**			
Not using	152 (93.8)	129 (94.2)	23 (92.0)
Using	10 (6.2)	8 (5.8)	2 (8.0)

Legend/key: *p<0.05, HSI = heaviness of smoking index, EC = e-cigarette, GCSE = General Certificate of Education

Percentages calculated from the total number of responders for each variable as not all questions had complete data

Prevalence of PPRS

35.8% (n=49, 95% CI 28-44%) of women reported PPRS.

In univariate analysis (Table 2), breastfeeding at 12 weeks postpartum was inversely associated with PPRS, whilst having a household member who was smoking postpartum was positively associated with PPRS. PPRS was also less common in those who used EC in pregnancy but not significantly so (p =0.1).

In multiple logistic regression, the significant independent predictors of PPRS were breastfeeding, use of ECs during pregnancy and household member smoking postpartum. The use of ECs during pregnancy (adjusted OR 0.34, 95% CI 0.13-0.85) and breastfeeding (adjusted OR 0.06, 95% CI 0.02-0.24) were associated with lower

For peer review only

risk of PPRS and a household member smoking postpartum with higher risk of PPRS (adjusted OR 11.1, 95% CI 2.47-50.2).

For peer review only

For peer review only - http://bmjopen.bmj.com/site/about/guidelines.xhtml

Table 2. Univariate and multiple logistic regression showing associations with postpartum return to smoking

Characteristics	All (women who quit smoking in pregnancy) (N= 162) N (col %)	% Returning to smoking postpartum (N=49) N (row %)	Crude OR (95% CI)	Adjusted OR (95% CI)
**Baseline/pregnancy variables**				
**Baseline smoking status**				
Current smoker	29 (17.9)	7 (24.1)	0.88 (0.33-2.35)	
Ex-smoker	133 (82.1)	42 (31.6)	Reference	
**Education**				
No qualifications	11 (6.8)	2 (18.2)	Reference	
GCSEs or above	150 (93.2)	46 (30.7)	1.39 (0.26-7.43)	
**Maternal age**				
Continuous – mean (SD)	26.9 (5.6)	26.7 (4.8)	0.98 (0.92-1.05)	
**Previous pregnancy**				
No previous pregnancy	73 (45.6)	25 (34.2)	Reference	
Previous pregnancy	87 (54.4)	23 (26.4)	0.73 (0.36-1.48)	
**NRT use in pregnancy**				
Not used NRT	129 (94.2)	47 (36.4)	Reference	
Used NRT	8 (5.8)	2 (25.0)	0.58 (0.11-3.00)	
**Confidence in staying smoke free (baseline)**				
Not at all-a little	20 (12.7)	8 (40.0)	Reference	
Moderately-extremely	137 (87.3)	38 (27.7)	0.62 (0.22-1.69)	
**Accessed support from SSS in pregnancy**				
Not accessed support	128 (93.4)	47 (36.7)	Reference	
Accessed support	9 (6.6)	2 (22.2)	0.49 (0.10-2.47)	
**EC use in pregnancy**□				
Never used EC in pregnancy	114 (70.4)	38 (33.3)	Reference	Reference
Used EC at some point during pregnancy	48 (29.6)	11 (22.9)	0.53 (0.24-1.19)	0.34 (0.13-0.85)*
**Postpartum**				
**Breastfeeding**				
Baby no longer/never breastfed	90 (66.2)	43 (47.8)	Reference	Reference
Baby still breastfeeding at 12 weeks postpartum	46 (33.8)	5 (10.9)	0.13 (0.05-0.37)**	0.06 (0.02-0.24)**
**Household member smoking postpartum**				
No household member smoking	120 (87.6)	39 (32.5)	Reference	
Household member smokes	17 (12.4)	10 (58.8)	2.97 (1.05-8.38)*	11.1 (2.47-50.2)**
**EC use postpartum**□				
Never used EC postpartum	109 (80.2)	38 (34.9)	Reference	
Used EC at some point postpartum	27 (19.9)	10 (37.0)	1.10 (0.46-2.64)	

EC = electronic cigarette, NRT = nicotine replacement therapy, GCSE = General Certificate of Secondary Education

□ No correlation between EC use in pregnancy and EC use postpartum detected

Variables that do not total 162 are missing

*p<0.05, **p<0.001

For peer review only

For peer review only - http://bmjopen.bmj.com/site/about/guidelines.xhtml

DISCUSSION

Key findings

This is the first study, to our knowledge, to explore use of ECs in pregnancy and postpartum, among other potential risk factors, for return to smoking postpartum. Using ECs in pregnancy, breastfeeding and not having a household member who smokes are associated with a lower likelihood of returning to smoking postpartum.

Strengths and weaknesses

The cohort collected longitudinal, prospective data on women's smoking behaviour during pregnancy and postpartum, reducing recall error. The cohort is likely to be broadly representative as the demographic profile of smokers is similar to other UK pregnancy cohorts[14], and the characteristics of the subsample included in our analyses reflect those known to be associated with smoking cessation in pregnancy[19].

A limitation is that the primary study was not designed to answer the research question; consequently, relatively small numbers of women reported returning to smoking postpartum and using ECs. This could limit the reliability of our findings; however, in previous research, not breastfeeding and household member smoking are commonly associated with postpartum return to smoking[11], suggesting our sample size was sufficient to detect some potentially relevant associations, but it is possible that more subtle ones were missed. We measured HSI, which was low in the majority of women; postpartum return to smoking only occurred in those with low HSI so we could not look at its independent effect. Reliance on self-reported smoking and EC use may lead to underreporting, particularly due to social stigma[20 21]. However, the surveys were anonymous, baseline surveys were completed discreetly in clinic and follow-ups were conducted in the privacy of home. As smoking prevalence varies internationally[22], the prevalence of EC use is also likely to vary, and these findings may only be generalisable to countries like the UK, where ECs are encouraged for smoking cessation. This cohort study was conducted in 2017-2018, since which time EC awareness, marketing and health advice may have

For peer review only - http://bmjopen.bmj.com/site/about/guidelines.xhtml

changed. However, as there are no similar published analyses on this topic our findings are an important contribution to the scientific literature.

Findings in context of previous literature

Our findings are consistent with previous studies in which less postpartum return to smoking was observed among mothers who breastfed and did not live with smokers[11]. To our knowledge, this is the first time that an association between EC use in pregnancy and postpartum return to smoking has been observed and the reason for this inverse relationship is not immediately clear.

Previous literature suggests that EC use, smoking and quitting may influence each other. Qualitative research has reported that one of the primary motivations for using ECs in pregnancy and postpartum is to quit smoking or prevent returning to smoking[23] In non-pregnant populations, a meta-analysis of three studies found that risk of relapse was higher among former smokers who used ECs (RR 1.38, 95% CI: 1.11–1.65), although no relationship was observed between relapse and frequency of EC use [24]. Conversely, qualitative research with non-pregnant former smokers observes that using ECs can support smoking relapse prevention by offering a long-term alternative to tobacco, and can prevent a smoking lapse becoming a full relapse[25]. Using ECs to avoid returning to smoking may be the reason behind EC use in pregnancy in our study, however we would therefore also expect to see a similar relationship for postpartum EC use. It may be that our sample size was too small to detect this association, or it is possible that other, non-measured confounders are responsible for our findings. Future research is needed to confirm the relationship between EC use and reduced postpartum return to smoking and establish whether this association could be causal.

Conclusion

We believe this is the first demonstration that EC use in pregnancy could influence postpartum return to smoking. Further research is needed to confirm this finding and

For peer review only - http://bmjopen.bmj.com/site/about/guidelines.xhtml

investigate the possibility that ECs could be used to prevent returning to smoking in the postpartum.

AUTHOR CONTRIBUTIONS

Conceptualization, SO, TC, MU, SC, SL; methodology, SO, TC, MU, SC, SL; formal analysis, LT, LL, SO; writing—original draft preparation, SO, LT, LL; writing—review and editing, SO, LT, LL, TC, MU, SC, SL; supervision, SO, TC, SC, SL, MU; project administration, SO, LT, LL; funding acquisition, SO, TC, SC, SL, MU. All authors have read and agreed to the published version of the manuscript.

FUNDING

This study/project is funded by the National Institute for Health Research (NIHR) School for Primary Care Research (NIHR SPCR -2016-102). The views expressed are those of the author(s) and not necessarily those of the NIHR or the Department of Health and Social Care. Prof Coleman is an NIHR Senior Investigator.

The original cohort study was funded by Cancer Research UK, Tobacco Advisory Group Project (Grant number C53479/A22733).

DATA SHARING

All data sharing requests can be made to SC (Principal Investigator to the original cohort).

COMPETING INTERESTS

The authors have no competing interests.

For peer review only

REFERENCES

1. NHS Digital. NHS Maternity Statistics, England 2019-20. Booking appointments. 2020 [Available from: https://digital.nhs.uk/data-and-information/publications/statistical/nhs-maternity-statistics/2019-20/booking-appointments, accessed 29.06.2021.
2. Taylor AE, Howe LD, Heron JE, et al. Maternal smoking during pregnancy and offspring smoking initiation: assessing the role of intrauterine exposure. *Addiction* 2014;109(6):1013-21. doi: https://doi.org/10.1111/add.12514
3. Hajek P, West R, Lee A, et al. Randomized controlled trial of a midwife-delivered brief smoking cessation intervention in pregnancy. *Addiction* 2001;96(3):485-94. doi: 10.1080/0965214002005446
4. Fingerhut LA, Kleinman JC, Kendrick JS. Smoking before, during, and after pregnancy. *Am J Public Health* 1990;80(5):541-44. doi: 10.2105/Ajph.80.5.541
5. Harmer C, Memon A. Factors associated with smoking relapse in the postpartum period: an analysis of the Child Health Surveillance System Data in southeast England. *Nicotine Tob Res* 2012;15(5):904-9. doi: 10.1093/ntr/nts221
6. National Center for Chronic Disease P, Health Promotion Office on S, Health. Reports of the Surgeon General. The Health Consequences of Smoking—50 Years of Progress: A Report of the Surgeon General. Atlanta (GA): Centers for Disease Control and Prevention (US) 2014.
7. Sims M, Tomkins S, Judge K, et al. Trends in and predictors of second-hand smoke exposure indexed by cotinine in children in England from 1996 to 2006. *Addiction* 2010;105(3):543-53. doi: 10.1111/j.1360-0443.2009.02805.x
8. Royal College of Physicians. *Passive smoking and children. A report of the Tobacco Advisory Group of the Royal College of Physicians* London2010 [Available from: https://www.rcplondon.ac.uk/sites/default/files/documents/passive-smoking-and-children.pdf, accessed 12.01.2015.
9. Leonardi-Bee J, Jere ML, Britton J. Exposure to parental and sibling smoking and the risk of smoking uptake in childhood and adolescence: a systematic review and meta-analysis. *Thorax* 2011;66(10):847-55. doi: 10.1136/thx.2010.153379
10. Livingstone-Banks J, Norris E, Hartmann-Boyce J, et al. Relapse prevention interventions for smoking cessation. *Cochrane Database Syst Rev* 2019(10) doi: 10.1002/14651858.CD003999.pub6
11. Orton S, Coleman T, Coleman-Haynes T, et al. Predictors of Postpartum Return to Smoking: A Systematic Review. *Nicotine Tob Res* 2017;20(6):665-73. doi: 10.1093/ntr/ntx163
12. West R, Beard E, Kale D, et al. Trends in electronic cigarette use in England 2021 [Available from: https://smokinginengland.info/graphs/e-cigarettes-latest-trends, accessed 08.07.2021.
13. Hartmann-Boyce J, McRobbie H, Butler AR, et al. Electronic cigarettes for smoking cessation. *Cochrane Database Syst Rev* 2021(4) doi: 10.1002/14651858.CD010216.pub5
14. Bowker K, Lewis S, Phillips L, et al. Pregnant women's use of e-cigarettes in the UK: a cross-sectional survey. *BJOG: An International Journal of Obstetrics & Gynaecology* 2020
15. Johnston EJ, Campbell K, Coleman T, et al. Safety of Electronic Cigarette Use During Breastfeeding: Qualitative Study Using Online Forum Discussions. *J Med Internet Res* 2019;21(8):e11506. doi: 10.2196/11506 [published Online First: 2019/08/14]
16. von Elm E, Altman DG, Egger M, et al. The Strengthening the Reporting of Observational Studies in Epidemiology (STROBE) statement: guidelines for reporting observational studies. *PLoS Med* 2007;4(10):e296. doi: 10.1371/journal.pmed.0040296 [published Online First: 2007/10/19]
17. Borland R, Yong HH, O'Connor RJ, et al. The reliability and predictive validity of the Heaviness of Smoking Index and its two components: findings from the International

For peer review only

Tobacco Control Four Country study. *Nicotine Tob Res* 2010;12 Suppl:S45-50. doi: 10.1093/ntr/ntq038 [published Online First: 2010/10/15]

18. Stata Statistical Software: Release 15 [program]: College Station, TX: StataCorp LLC, 2017.
19. Riaz M, Lewis S, Naughton F, et al. Predictors of smoking cessation during pregnancy: a systematic review and meta-analysis. *Addiction* 2018;113(4):610-22.
20. Bowker K, Orton S, Cooper S, et al. Views on and experiences of electronic cigarettes: a qualitative study of women who are pregnant or have recently given birth. *BMC Pregnancy and Childbirth* 2018;18(1):233. doi: ARTN 233

10.1186/s12884-018-1856-4

21. Grant A, Morgan M, Gallagher D, et al. Smoking during pregnancy, stigma and secrets: Visual methods exploration in the UK. *Women and Birth* 2020;33(1):70-76.
22. Lange S, Probst C, Rehm J, et al. National, regional, and global prevalence of smoking during pregnancy in the general population: a systematic review and meta-analysis. *The Lancet Global Health* 2018;6(7):e769-e76.
23. McNeill A, Brose LS, Calder R, et al. Vaping in England: an evidence update including mental health and pregnancy, March 2020: a report commissioned by Public Health England. London: Public Health England, 2020.
24. Barufaldi LA, Guerra RL, de Albuquerque RdCR, et al. Risk of smoking relapse with the use of electronic cigarettes: A systematic review with meta-analysis of longitudinal studies. *Tob Prev Cessat* 2021;29:29-29. doi: 10.18332/tpc/132964
25. Notley C, Ward E, Dawkins L, et al. The unique contribution of e-cigarettes for tobacco harm reduction in supporting smoking relapse prevention. *Harm Reduct J* 2018;15(1):31. doi: 10.1186/s12954-018-0237-7

For peer review only

Figure S1 – Consort diagram NB: five self-reported non-smokers from FU1 for PLS National were removed from n=167 as they quit more than 3 months before pregnancy

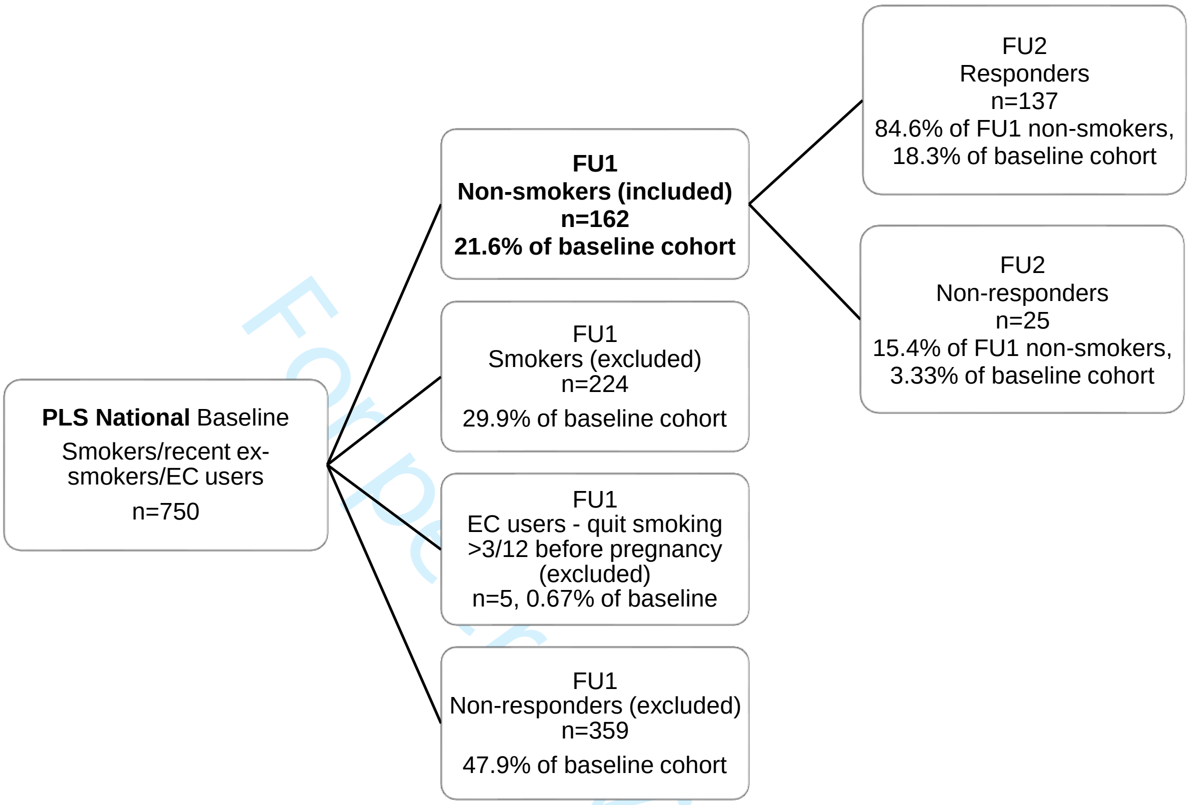

STROBE 2007 (v4) Statement—Checklist of items that should be included in reports of *cohort studies*

Section/Topic	Item #	Recommendation	Reported on page #
**Title and abstract**	1	(*a*) Indicate the study's design with a commonly used term in the title or the abstract	2
		(*b*) Provide in the abstract an informative and balanced summary of what was done and what was found	2
**Introduction**			
Background/rationale	2	Explain the scientific background and rationale for the investigation being reported	3
Objectives	3	State specific objectives, including any prespecified hypotheses	3-4
**Methods**			
Study design	4	Present key elements of study design early in the paper	5
Setting	5	Describe the setting, locations, and relevant dates, including periods of recruitment, exposure, follow-up, and data collection	5
Participants	6	(*a*) Give the eligibility criteria, and the sources and methods of selection of participants. Describe methods of follow-up	5
		(*b*) For matched studies, give matching criteria and number of exposed and unexposed	n/a
Variables	7	Clearly define all outcomes, exposures, predictors, potential confounders, and effect modifiers. Give diagnostic criteria, if applicable	6
Data sources/ measurement	8*	For each variable of interest, give sources of data and details of methods of assessment (measurement). Describe comparability of assessment methods if there is more than one group	6
Bias	9	Describe any efforts to address potential sources of bias	5
Study size	10	Explain how the study size was arrived at	5
Quantitative variables	11	Explain how quantitative variables were handled in the analyses. If applicable, describe which groupings were chosen and why	5-6
Statistical methods	12	(*a*) Describe all statistical methods, including those used to control for confounding	6-7
		(*b*) Describe any methods used to examine subgroups and interactions	n/a
		(*c*) Explain how missing data were addressed	6
		(*d*) If applicable, explain how loss to follow-up was addressed	6-7
		(*e*) Describe any sensitivity analyses	n/a
**Results**			

For peer review only

Participants	13*	(a) Report numbers of individuals at each stage of study—eg numbers potentially eligible, examined for eligibility, confirmed eligible, included in the study, completing follow-up, and analysed	S1
		(b) Give reasons for non-participation at each stage	n/a, non-response to survey
		(c) Consider use of a flow diagram	S1
Descriptive data	14*	(a) Give characteristics of study participants (eg demographic, clinical, social) and information on exposures and potential confounders	Table 1 & 2
		(b) Indicate number of participants with missing data for each variable of interest	Table 2 footnote
		(c) Summarise follow-up time (eg, average and total amount)	5
Outcome data	15*	Report numbers of outcome events or summary measures over time	7-10
Main results	16	(*a*) Give unadjusted estimates and, if applicable, confounder-adjusted estimates and their precision (eg, 95% confidence interval). Make clear which confounders were adjusted for and why they were included	7-10
		(*b*) Report category boundaries when continuous variables were categorized	Table 1 & 2
		(*c*) If relevant, consider translating estimates of relative risk into absolute risk for a meaningful time period	n/a
Other analyses	17	Report other analyses done—eg analyses of subgroups and interactions, and sensitivity analyses	6-7
**Discussion**			
Key results	18	Summarise key results with reference to study objectives	11
**Limitations**			
Interpretation	20	Give a cautious overall interpretation of results considering objectives, limitations, multiplicity of analyses, results from similar studies, and other relevant evidence	11-12
Generalisability	21	Discuss the generalisability (external validity) of the study results	11-12
**Other information**			
Funding	22	Give the source of funding and the role of the funders for the present study and, if applicable, for the original study on which the present article is based	13

*Give information separately for cases and controls in case-control studies and, if applicable, for exposed and unexposed groups in cohort and cross-sectional studies.

Note: An Explanation and Elaboration article discusses each checklist item and gives methodological background and published examples of transparent reporting. The STROBE checklist is best used in conjunction with this article (freely available on the Web sites of PLoS Medicine at http://www.plosmedicine.org/, Annals of Internal Medicine at http://www.annals.org/, and Epidemiology at http://www.epidem.com/). Information on the STROBE Initiative is available at www.strobe-statement.org.

For peer review only

ARE E-CIGARETTES ASSOCIATED WITH POSTPARTUM RETURN TO SMOKING? SECONDARY ANALYSES OF A UK PREGNANCY LONGITUDINAL COHORT

Journal:	*BMJ Open*
Manuscript ID	bmjopen-2022-061028.R1
Article Type:	Original research
Date Submitted by the Author:	10-Mar-2022

Complete List of Authors:	Orton, Sophie; University of Nottingham<br>Taylor, Lauren; University of Nottingham, School of Medicine<br>Laing, Libby; University of Nottingham, School of Medicine<br>Lewis, Sarah; University of Nottingham, School of Medicine<br>Ussher, Michael; St George's University of London, Division of Population Health Sciences and Education; University of Stirling Institute for Social Marketing and Health<br>Coleman, Tim; University of Nottingham, School of Medicine<br>Cooper, Sue; University of Nottingham, School of Medicine

**Primary Subject Heading**:	Smoking and tobacco
Secondary Subject Heading:	Addiction, Public health
Keywords:	PRIMARY CARE, PREVENTIVE MEDICINE, PUBLIC HEALTH, REPRODUCTIVE MEDICINE

SCHOLARONE™
Manuscripts

I, the Submitting Author has the right to grant and does grant on behalf of all authors of the Work (as defined in the below author licence), an exclusive licence and/or a non-exclusive licence for contributions from authors who are: i) UK Crown employees; ii) where BMJ has agreed a CC-BY licence shall apply, and/or iii) in accordance with the terms applicable for US Federal Government officers or employees acting as part of their official duties; on a worldwide, perpetual, irrevocable, royalty-free basis to BMJ Publishing Group Ltd ("BMJ") its licensees and where the relevant Journal is co-owned by BMJ to the co-owners of the Journal, to publish the Work in this journal and any other BMJ products and to exploit all rights, as set out in our licence.

The Submitting Author accepts and understands that any supply made under these terms is made by BMJ to the Submitting Author unless you are acting as an employee on behalf of your employer or a postgraduate student of an affiliated institution which is paying any applicable article publishing charge ("APC") for Open Access articles. Where the Submitting Author wishes to make the Work available on an Open Access basis (and intends to pay the relevant APC), the terms of reuse of such Open Access shall be governed by a Creative Commons licence – details of these licences and which Creative Commons licence will apply to this Work are set out in our licence referred to above.

Other than as permitted in any relevant BMJ Author's Self Archiving Policies, I confirm this Work has not been accepted for publication elsewhere, is not being considered for publication elsewhere and does not duplicate material already published. I confirm all authors consent to publication of this Work and authorise the granting of this licence.

For peer review only

ARE E-CIGARETTES ASSOCIATED WITH POSTPARTUM RETURN TO SMOKING? SECONDARY ANALYSES OF A UK PREGNANCY LONGITUDINAL COHORT

Sophie Orton PhD[1*], Lauren Taylor BMBS[1], Libby Laing PhD[1], Sarah Lewis Prof[1], Michael Ussher Prof[2, 3], Tim Coleman Prof[1], Sue Cooper PhD[1]

1. School of Medicine, University of Nottingham, Nottingham, UK

2. Population Health Research Institute, St George's, University of London, London SW17 0RE, UK.

3. Institute for Social Marketing and Health, University of Stirling, Stirling FK9 4LA, UK.

* Corresponding author: Dr Sophie Orton, University of Nottingham, Sophie.orton@nottingham.ac.uk

Word count: 1996

For peer review only - http://bmjopen.bmj.com/site/about/guidelines.xhtml

ABSTRACT

Objectives: Postpartum return to smoking (PPRS) is an important public health problem. E-cigarette (EC) use has increased in recent years, and in a contemporary UK pregnancy cohort, we investigated factors, including ECs use, associated with PPRS.

Design: Secondary analyses of a longitudinal cohort survey with questionnaires at baseline (8–26 weeks' gestation), late pregnancy (34–36 weeks) and 3 months after delivery.

Setting: 17 hospitals in England and Scotland in 2017.

Participants: The cohort recruited 750 women who were current or recent ex-smokers and/or EC users. A subgroup of women reported being abstinent from smoking in late pregnancy (n=162, 21.6%), and of these 137 (84.6%) completed the postpartum questionnaire and were included in analyses.

Outcome measures: Demographics, smoking behaviours and beliefs, views and experience of ECs and infant feeding.

Results: 35.8% (95% CI 28-44%) of women reported PPRS. EC use in pregnancy (adjusted OR 0.34, 95% CI 0.13-0.85) and breastfeeding (adjusted OR 0.06, 95% CI 0.02-0.24) were inversely associated with PPRS, whilst household member smoking at 3 months postpartum was positively associated with PPRS (adjusted OR 11.1, 95% CI 2.47-50.2).

Conclusion: EC use in pregnancy could influence postpartum return to smoking. Further research is needed to confirm this and investigate whether ECs could be used to prevent PPRS.

For peer review only - http://bmjopen.bmj.com/site/about/guidelines.xhtml

Strengths and limitations of this study

- This is the first study, to our knowledge, to explore use of ECs in pregnancy and postpartum, among other potential risk factors, for return to smoking postpartum.
- The cohort collected longitudinal, prospective data on women's smoking behaviour during pregnancy and postpartum, reducing recall error.
- The cohort is likely to be broadly representative as the demographic profile of smokers is similar to other UK pregnancy cohorts.
- The primary study was not designed to answer the research question; consequently, relatively small numbers of women reported returning to smoking postpartum and using ECs.
- Smoking and EC use were self-reported, which could lead to underreporting due to social stigma; however the surveys were completed anonymously which may have minimised this issue.

For peer review only

INTRODUCTION

Helping pregnant women quit smoking and remain abstinent in the long term is an important public health issue. In 2019/2020 12.1% of women in England were smoking in early pregnancy[1], however around half attempt cessation after conception[2]. Unfortunately, relapse is common, with up to 75% returning to smoking within 12 months of giving birth[3-5]. Reducing maternal postpartum return to smoking (PPRS) would have substantial health benefits for both mother and baby[6]; women would reduce their own risks of smoking related illness, reduce ill health and mortality associated with second hand smoke exposure among their children[7 8], and reduce the likelihood of their children becoming smokers themselves in later life[9].

There are currently no effective interventions for reducing PPRS[10]. Understanding the factors that are associated with PPRS is essential for the development of targeted and evidence-based interventions. A systematic review[11] observed that women that returned to smoking postpartum tended to be less well educated, younger, multiparous, living with a partner or household member who smoked, experiencing higher stress, depression or anxiety, not breastfeeding, intending to quit only for pregnancy, and having low confidence in remaining quit after giving birth.

Since this review, e-cigarettes (ECs) have become increasingly popular [12]. ECs have potential for public health benefit as they do not involve tobacco combustion, which is the main source of harm from conventional cigarettes. In non-pregnant smokers, there is moderate certainty that ECs containing nicotine improve cessation rates, compared with non-nicotine vaping products or nicotine replacement therapy (NRT)[13]. In 2021, ECs were used in 29% of UK quit attempts[12] and around 5% of UK women report using ECs in pregnancy[14]. There is also evidence from qualitative studies that some women are using ECs to avoid returning to smoking after having their baby[15]. Hence, using a contemporary UK pregnancy cohort, we investigated factors associated with PPRS, including EC use in pregnancy and afterwards[11].

For peer review only - http://bmjopen.bmj.com/site/about/guidelines.xhtml

METHODS

This paper follows the STROBE reporting guidelines[16] (S1). We conducted secondary analyses on data collected in a UK longitudinal pregnancy cohort designed to explore the use of and attitudes towards ECs during pregnancy and postpartum. Full methods and cohort characteristics are presented elsewhere[14]. In brief, the cohort recruited women from 17 hospital antenatal clinics from June to November 2017. Hospitals had varying smoking in pregnancy rates and were from a range of geographical locations in England and Scotland. Screening surveys were systematically handed out to all pregnant women attending selected antenatal clinics. Women were eligible to join the cohort if they self-reported being either current or recent ex-smokers (quit smoking in the 3 months prior to pregnancy), and/or current EC users. Data were collected at 8-26 weeks gestation (baseline), 34–36 weeks gestation (follow-up one, FU1) and 12 weeks postpartum (follow-up two, FU2). Baseline questionnaires asked about maternal demographics (age, education, ethnicity), parity, smoking behaviours and beliefs, and use of ECs. FU1 and FU2 questionnaires were similar to baseline but also asked about plans for infant feeding, how baby was being fed and smoking/EC use postpartum.

Patient and Public Involvement

Patient and public involvement colleagues contributed to the design of the study, including providing feedback on the importance of the research question, designing study materials and advising on study recruitment.

Ethics approval

Ethical approval was granted by the South West Frenchay Research Ethics Committee (IRAS project ID 222886).

Selection of respondents for inclusion in analyses

Women who reported having quit smoking either in the 3 months prior to pregnancy, or during pregnancy, and were still abstinent by FU1 (34-36 weeks gestation) were included in the analysis.

For peer review only - http://bmjopen.bmj.com/site/about/guidelines.xhtml

Dependant and explanatory variables

The primary outcome measure was return to smoking at 3 months postpartum using participant's responses to the question 'which statement best describes your smoking right now?'. Response options were 'I do not smoke at all' 'I smoke occasionally, but not every day', 'I smoke every day, but less than when I was pregnant', 'I smoke every day, about the same as when I was pregnant' and 'I smoke every day and tend to smoke more than when I was pregnant'. Women who reported smoking at least occasionally were considered to have returned to smoking.

The explanatory variables measured at baseline/during pregnancy were: baseline smoking status (current smoker or recent ex-smoker), education ('no qualifications' or 'General Certificate of Secondary Education or above', using highest educational qualification), maternal age, previous pregnancies (no previous pregnancy' or 'previous pregnancy'), baseline Heaviness of Smoking Index (HSI) ('low', 'moderate' or 'high')[17], NRT use in pregnancy ('not used NRT' or 'used NRT'), confidence in staying smoke free ('not at all – a little' or 'moderately – extremely'), whether they had accessed stop smoking support during pregnancy ('not accessed'' or 'accessed'), and EC use during pregnancy ('never used EC in pregnancy' or 'used EC at some point during pregnancy'). Postpartum explanatory variables were breastfeeding ('baby no longer/never breastfed' or 'baby still breastfeeding at 12 weeks postpartum'), household member smoking ('no household member smoking' or 'household member smokes' and EC use ('never used EC postpartum' or 'used EC at some point postpartum').

Analysis

Analyses were conducted using Stata version 13.1[18]. We handled missing data using available case analysis. Baseline demographic characteristics were analysed using descriptive statistics. Prevalence of PPRS was calculated for those who responded at FU2.

For peer review only - http://bmjopen.bmj.com/site/about/guidelines.xhtml

An exploratory logistic regression was conducted to identify factors associated with PPRS. Collinearity between variables was assessed using correlation analysis and no significant correlations were identified. HSI was excluded from logistic regression because all those in the PPRS group had low HSI. We used an iterative approach to determine which variables best predicted PRRS. Variables were selected based on those identified to be associated with PPRS in previous literature[11], and those of theoretical interest (e.g. EC use). We first determined which of these variables were significantly associated ($p < 0.05$) with PPRS in univariate logistic regression. These variables were then included together in a multivariate logistic regression model. Variables that were not significant were removed from the model, so that only significant variables ($p < 0.05$) were retained. Variables which had been excluded, including those that were not significant in univariate analysis, were added into the model consecutively to assess whether they became significant and retained if so. Effects were expressed as odds ratios and 95% confidence intervals, and likelihood ratio test p-values ($p< .05$) are presented.

RESULTS

Response rates

The total cohort included 750 women, and 162 (21.6%) were eligible and included in analyses. From these eligible women, response rate for the FU2 (postpartum) survey was 84.6% (137/162) (Figure S1).

Cohort characteristics

Table 1 presents the baseline characteristics of women who were abstinent from smoking in late pregnancy (i.e., at FU1) and of respondents and non-respondents to the postpartum questionnaire (FU2). At baseline, 17.9% smoked, 82.4% had low addiction levels and 93.8% were not using ECs. 29.6% used an EC at some point in pregnancy, and 19.9% used an EC at some point in the 12 weeks since having their baby. Those who did not respond to FU2 were more likely to be a current smoker at baseline and have a lower educational level.

For peer review only - http://bmjopen.bmj.com/site/about/guidelines.xhtml

Table 1: Baseline questionnaire characteristics of those participants who were non-smokers in late pregnancy and a comparison between those who did and did not respond to the postpartum survey

Characteristics at baseline (8-26 weeks gestation)	Women abstinent from smoking in late pregnancy N (%) N= 162	Responded to postpartum questionnaire N (%) N=137	Did not respond to postpartum questionnaire N (%) N=25
**Smoking status**			
Recent ex-smoker	133 (82.1)	116 (84.7)	17 (68.0)
Current smoker	29 (17.9)	21 (15.3)	8 (32.0)*
**HSI score**			
Low addiction	28 (82.4)	22 (84.6)	6 (75.0)
Moderate addiction	6 (17.7)	4 (15.4)	2 (25.0)
High addiction	0 (0.0)	0 (0.0)	0 (0.0)
**Education**			
No qualifications	11 (6.8)	7 (5.15)	4 (16.0)
GCSE or above (including other)	150 (93.2)	129 (94.9)	21 (84.0)*
**Maternal age (Mean, SD)**	26.9 (5.6)	27.1 (5.3)	25.9 (7.0)
**Ethnicity**			
White British	139 (86.3)	118 (86.8)	21 (84.0)
Other ethnicity	22 (13.7)	18 (13.2)	4 (16.0)
**Previous pregnancy**			
No previous pregnancy	73 (45.6)	64 (47.1)	9 (37.5)
Previous pregnancy	87 (54.4)	72 (52.9)	15 (62.5)
**Confidence in staying smoke free**			
Not at all-a little	20 (12.7)	18 (13.5)	2 (8.33)
Moderately-extremely	137 (87.3)	115 (86.5)	22 (91.7)
**EC use**			
Not using	152 (93.8)	129 (94.2)	23 (92.0)
Using	10 (6.2)	8 (5.8)	2 (8.0)

Legend/key: *p<0.05, HSI = heaviness of smoking index, EC = e-cigarette, GCSE = General Certificate of Education

Percentages calculated from the total number of responders for each variable as not all questions had complete data

For peer review only

Prevalence of PPRS

35.8% (n=49, 95% CI 28-44%) of women reported PPRS.

In univariate analysis (Table 2), breastfeeding at 12 weeks postpartum was inversely associated with PPRS, whilst having a household member who was smoking postpartum was positively associated with PPRS. PPRS was also less common in those who used EC in pregnancy but not significantly so (p =0.1).

For peer review only - http://bmjopen.bmj.com/site/about/guidelines.xhtml

In multiple logistic regression, the significant independent predictors of PPRS were breastfeeding, use of ECs during pregnancy and household member smoking postpartum. The use of ECs during pregnancy (adjusted OR 0.34, 95% CI 0.13-0.85) and breastfeeding (adjusted OR 0.06, 95% CI 0.02-0.24) were associated with lower risk of PPRS and a household member smoking postpartum with higher risk of PPRS (adjusted OR 11.1, 95% CI 2.47-50.2).

E-cigarette use

42 (30.7%) reported using an EC at some point in pregnancy, and 27 (19.9%) reported using an EC at some point postpartum. 20 (48.8%) of those who used an EC in pregnancy also used an EC postpartum.

For peer review only

Table 2. Univariate and multiple logistic regression showing associations with postpartum return to smoking

**Characteristics**	**All (women who quit smoking in pregnancy and completed FU2) (N= 137) N**	**% Returning to smoking postpartum (N=49) N (row %)**	**Crude OR (95% CI)**	**Adjusted OR (95% CI) N=136**
**Baseline/pregnancy variables**				
**Baseline smoking status**				
Current smoker	21	7 (33.3)	0.88 (0.33-2.35)	
Ex-smoker	116	42 (36.2)	Reference	
**Education**				
No qualifications	7	2 (28.6)	Reference	
GCSEs or above	129	46 (35.7)	1.39 (0.26-7.43)	
**Maternal age**				
Continuous – mean (SD)	27.1	26.7 (4.8)	0.98 (0.92-1.05)	
**Previous pregnancy**				
No previous pregnancy	64	25 (39.1)	Reference	
Previous pregnancy	72	23 (31.9)	0.73 (0.36-1.48)	
**Confidence in staying smoke free (baseline)**				
Not at all-a little	18	8 (44.4)	Reference	
Moderately-extremely	115	38 (33.0)	0.62 (0.22-1.69)	
**NRT use in pregnancy** [a]				
Not used NRT	129	47 (36.4)	Reference	
Used NRT	8	2 (25.0)	0.58 (0.11-3.00)	
**Accessed support from SSS in pregnancy** [a]				
Not accessed support	128	47 (36.7)	Reference	
Accessed support	9	2 (22.2)	0.49 (0.10-2.47)	
**EC use in pregnancy** [a]				
Never used EC in pregnancy	95	38 (40.0)	Reference	Reference
Used EC at some point during pregnancy	42	11 (26.2)	0.53 (0.24-1.19)	0.34 (0.13-0.85)*
**Postpartum**				
**Breastfeeding**				
Baby no longer/never breastfed	90	43 (47.8)	Reference	Reference
Baby still breastfeeding at 12 weeks postpartum	46	5 (10.9)	0.13 (0.05-0.37)**	0.06 (0.02-0.24)**
**Household member smoking postpartum**				
No household member smoking	120	39 (32.5)	Reference	
Household member smokes	17	10 (58.8)	2.97 (1.05-8.38)*	11.1 (2.47-50.2)**
**EC use postpartum**				
Never used EC postpartum	109	38 (34.9)	Reference	
Used EC at some point postpartum	27	10 (37.0)	1.10 (0.46-2.64)	

EC = electronic cigarette, NRT = nicotine replacement therapy, GCSE = General Certificate of Secondary Education

For peer review only

For peer review only - http://bmjopen.bmj.com/site/about/guidelines.xhtml

[a] Data collated from baseline and FU1

 No correlation between EC use in pregnancy and EC use postpartum detected

Variables that do not total 137 are missing

*p<0.05, **p<0.001

DISCUSSION

Key findings

This is the first study, to our knowledge, to explore use of ECs in pregnancy and postpartum, among other potential odds of postpartum return to smoking . Using ECs in pregnancy, breastfeeding and not having a household member who smokes are associated with a lower likelihood of returning to smoking postpartum.

Strengths and weaknesses

The cohort collected longitudinal, prospective data on women's smoking behaviour during pregnancy and postpartum, reducing recall error. The cohort is likely to be broadly representative as the demographic profile of smokers is similar to other UK pregnancy cohorts[14], and the characteristics of the subsample included in our analyses reflect those known to be associated with smoking cessation in pregnancy[19].

A limitation is that the primary study was not designed to answer the research question; consequently, relatively small numbers of women reported returning to smoking postpartum and using ECs. This could limit the reliability of our findings; however, in previous research, not breastfeeding and household member smoking are commonly associated with postpartum return to smoking[11], suggesting our sample size was sufficient to detect some potentially relevant associations, but it is possible that more subtle ones were missed. We measured HSI, which was low in the majority of women; postpartum return to smoking only occurred in those with low HSI so we could not look at its independent effect. Reliance on self-reported smoking and EC use may lead to underreporting, particularly due to social stigma[20 21]. However, the surveys were anonymous, baseline surveys were completed discreetly in clinic and follow-ups were conducted in the privacy of home. As

For peer review only - http://bmjopen.bmj.com/site/about/guidelines.xhtml

smoking prevalence varies internationally[22], the prevalence of EC use is also likely to vary, and these findings may only be generalisable to countries like the UK, where ECs are encouraged for smoking cessation. This cohort study was conducted in 2017-2018, since which time EC awareness, marketing and health advice may have changed. However, as there are no similar published analyses on this topic our findings are an important contribution to the scientific literature.

Findings in context of previous literature

Our findings are consistent with previous studies in which less postpartum return to smoking was observed among mothers who breastfed and did not live with smokers[11]. To our knowledge, this is the first time that an association between EC use in pregnancy and postpartum return to smoking has been observed and the reason for this inverse relationship is not immediately clear.

Previous literature suggests that EC use, smoking and quitting may influence each other. Qualitative research has reported that one of the primary motivations for using ECs in pregnancy and postpartum is to quit smoking or prevent returning to smoking[23]. In non-pregnant populations, a meta-analysis of three studies found that risk of relapse was higher among former smokers who used ECs (RR 1.38, 95% CI: 1.11–1.65), although no relationship was observed between relapse and frequency of EC use [24]. Conversely, qualitative research with non-pregnant former smokers observes that using ECs can support smoking relapse prevention by offering a long-term alternative to tobacco, and can prevent a smoking lapse becoming a full relapse[25]. Among ECs users in this cohort, the most commonly reported reasons for using ECs both in pregnancy and postpartum were to quit smoking and avoid returning to smoking[14]. We would therefore have expected to also see a similar relationship between postpartum EC use and postpartum return to smoking. It may be that our sample size was too small to detect this association, or it is possible that other, non-measured confounders are responsible for our findings. Future research is needed to confirm the relationship between EC use and reduced postpartum return to smoking and establish whether this association could be causal.

For peer review only - http://bmjopen.bmj.com/site/about/guidelines.xhtml

Conclusion

We believe this is the first demonstration that EC use in pregnancy could influence postpartum return to smoking. Further research is needed to confirm this finding and investigate the possibility that ECs could be used to prevent returning to smoking in the postpartum.

AUTHOR CONTRIBUTIONS

Conceptualization, SO, TC, MU, SC, SL; methodology, SO, TC, MU, SC, SL; formal analysis, LT, LL, SO; writing—original draft preparation, SO, LT, LL; writing—review and editing, SO, LT, LL, TC, MU, SC, SL; supervision, SO, TC, SC, SL, MU; project administration, SO, LT, LL; funding acquisition, SO, TC, SC, SL, MU. All authors have read and agreed to the published version of the manuscript.

FUNDING

This study/project is funded by the National Institute for Health Research (NIHR) School for Primary Care Research (NIHR SPCR -2016-102). The views expressed are those of the author(s) and not necessarily those of the NIHR or the Department of Health and Social Care. Prof Coleman is an NIHR Senior Investigator.

The original cohort study was funded by Cancer Research UK, Tobacco Advisory Group Project (Grant number C53479/A22733).

DATA SHARING

All data sharing requests can be made to SC (Principal Investigator to the original cohort).

COMPETING INTERESTS

The authors have no competing interests.

For peer review only - http://bmjopen.bmj.com/site/about/guidelines.xhtml

REFERENCES

1. NHS Digital. NHS Maternity Statistics, England 2019-20. Booking appointments. 2020 [Available from: https://digital.nhs.uk/data-and-information/publications/statistical/nhs-maternity-statistics/2019-20/booking-appointments, accessed 29.06.2021.
2. Taylor AE, Howe LD, Heron JE, et al. Maternal smoking during pregnancy and offspring smoking initiation: assessing the role of intrauterine exposure. *Addiction* 2014;109(6):1013-21. doi: https://doi.org/10.1111/add.12514
3. Hajek P, West R, Lee A, et al. Randomized controlled trial of a midwife-delivered brief smoking cessation intervention in pregnancy. *Addiction* 2001;96(3):485-94. doi: 10.1080/0965214002005446
4. Fingerhut LA, Kleinman JC, Kendrick JS. Smoking before, during, and after pregnancy. *Am J Public Health* 1990;80(5):541-44. doi: 10.2105/Ajph.80.5.541
5. Harmer C, Memon A. Factors associated with smoking relapse in the postpartum period: an analysis of the Child Health Surveillance System Data in southeast England. *Nicotine Tob Res* 2012;15(5):904-9. doi: 10.1093/ntr/nts221
6. National Center for Chronic Disease P, Health Promotion Office on S, Health. Reports of the Surgeon General. The Health Consequences of Smoking—50 Years of Progress: A Report of the Surgeon General. Atlanta (GA): Centers for Disease Control and Prevention (US) 2014.
7. Sims M, Tomkins S, Judge K, et al. Trends in and predictors of second-hand smoke exposure indexed by cotinine in children in England from 1996 to 2006. *Addiction* 2010;105(3):543-53. doi: 10.1111/j.1360-0443.2009.02805.x
8. Royal College of Physicians. *Passive smoking and children. A report of the Tobacco Advisory Group of the Royal College of Physicians* London2010 [Available from: https://www.rcplondon.ac.uk/sites/default/files/documents/passive-smoking-and-children.pdf, accessed 12.01.2015.
9. Leonardi-Bee J, Jere ML, Britton J. Exposure to parental and sibling smoking and the risk of smoking uptake in childhood and adolescence: a systematic review and meta-analysis. *Thorax* 2011;66(10):847-55. doi: 10.1136/thx.2010.153379
10. Livingstone-Banks J, Norris E, Hartmann-Boyce J, et al. Relapse prevention interventions for smoking cessation. *Cochrane Database Syst Rev* 2019(10) doi: 10.1002/14651858.CD003999.pub6
11. Orton S, Coleman T, Coleman-Haynes T, et al. Predictors of Postpartum Return to Smoking: A Systematic Review. *Nicotine Tob Res* 2017;20(6):665-73. doi: 10.1093/ntr/ntx163
12. West R, Beard E, Kale D, et al. Trends in electronic cigarette use in England 2021 [Available from: https://smokinginengland.info/graphs/e-cigarettes-latest-trends, accessed 08.07.2021.
13. Hartmann-Boyce J, McRobbie H, Butler AR, et al. Electronic cigarettes for smoking cessation. *Cochrane Database Syst Rev* 2021(4) doi: 10.1002/14651858.CD010216.pub5
14. Bowker K, Lewis S, Phillips L, et al. Pregnant women's use of e-cigarettes in the UK: a cross-sectional survey. *BJOG: An International Journal of Obstetrics & Gynaecology* 2020
15. Johnston EJ, Campbell K, Coleman T, et al. Safety of Electronic Cigarette Use During Breastfeeding: Qualitative Study Using Online Forum Discussions. *J Med Internet Res* 2019;21(8):e11506. doi: 10.2196/11506 [published Online First: 2019/08/14]
16. von Elm E, Altman DG, Egger M, et al. The Strengthening the Reporting of Observational Studies in Epidemiology (STROBE) statement: guidelines for reporting observational studies. *PLoS Med* 2007;4(10):e296. doi: 10.1371/journal.pmed.0040296 [published Online First: 2007/10/19]
17. Borland R, Yong HH, O'Connor RJ, et al. The reliability and predictive validity of the Heaviness of Smoking Index and its two components: findings from the International

For peer review only - http://bmjopen.bmj.com/site/about/guidelines.xhtml

Tobacco Control Four Country study. *Nicotine Tob Res* 2010;12 Suppl:S45-50. doi: 10.1093/ntr/ntq038 [published Online First: 2010/10/15]
18. Stata Statistical Software: Release 15 [program]: College Station, TX: StataCorp LLC, 2017.
19. Riaz M, Lewis S, Naughton F, et al. Predictors of smoking cessation during pregnancy: a systematic review and meta-analysis. *Addiction* 2018;113(4):610-22.
20. Bowker K, Orton S, Cooper S, et al. Views on and experiences of electronic cigarettes: a qualitative study of women who are pregnant or have recently given birth. *BMC Pregnancy and Childbirth* 2018;18(1):233. doi: ARTN 233

10.1186/s12884-018-1856-4
21. Grant A, Morgan M, Gallagher D, et al. Smoking during pregnancy, stigma and secrets: Visual methods exploration in the UK. *Women and Birth* 2020;33(1):70-76.
22. Lange S, Probst C, Rehm J, et al. National, regional, and global prevalence of smoking during pregnancy in the general population: a systematic review and meta-analysis. *The Lancet Global Health* 2018;6(7):e769-e76.
23. McNeill A, Brose LS, Calder R, et al. Vaping in England: an evidence update including mental health and pregnancy, March 2020: a report commissioned by Public Health England. London: Public Health England, 2020.
24. Barufaldi LA, Guerra RL, de Albuquerque RdCR, et al. Risk of smoking relapse with the use of electronic cigarettes: A systematic review with meta-analysis of longitudinal studies. *Tob Prev Cessat* 2021;29:29-29. doi: 10.18332/tpc/132964
25. Notley C, Ward E, Dawkins L, et al. The unique contribution of e-cigarettes for tobacco harm reduction in supporting smoking relapse prevention. *Harm Reduct J* 2018;15(1):31. doi: 10.1186/s12954-018-0237-7

For peer review only

For peer review only - http://bmjopen.bmj.com/site/about/guidelines.xhtml

Figure S1 – Consort diagram NB: five self-reported non-smokers from FU1 for PLS National were removed from n=167 as they quit more than 3 months before pregnancy

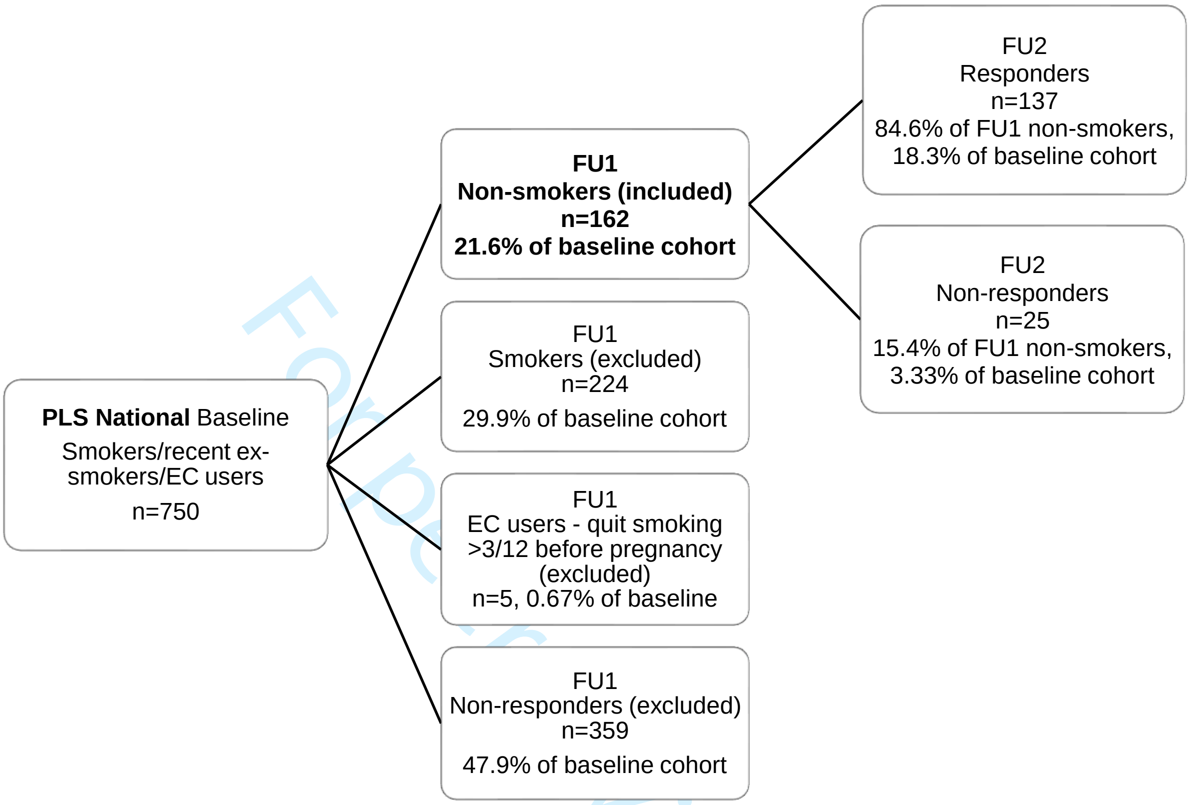

For peer review only

STROBE 2007 (v4) Statement—Checklist of items that should be included in reports of *cohort studies*

Section/Topic	Item #	Recommendation	Reported on page #
**Title and abstract**	1	(*a*) Indicate the study's design with a commonly used term in the title or the abstract	2
		(*b*) Provide in the abstract an informative and balanced summary of what was done and what was found	2
**Introduction**			
Background/rationale	2	Explain the scientific background and rationale for the investigation being reported	3
Objectives	3	State specific objectives, including any prespecified hypotheses	3-4
**Methods**			
Study design	4	Present key elements of study design early in the paper	5
Setting	5	Describe the setting, locations, and relevant dates, including periods of recruitment, exposure, follow-up, and data collection	5
Participants	6	(*a*) Give the eligibility criteria, and the sources and methods of selection of participants. Describe methods of follow-up	5
		(*b*) For matched studies, give matching criteria and number of exposed and unexposed	n/a
Variables	7	Clearly define all outcomes, exposures, predictors, potential confounders, and effect modifiers. Give diagnostic criteria, if applicable	6
Data sources/ measurement	8*	For each variable of interest, give sources of data and details of methods of assessment (measurement). Describe comparability of assessment methods if there is more than one group	6
Bias	9	Describe any efforts to address potential sources of bias	5
Study size	10	Explain how the study size was arrived at	5
Quantitative variables	11	Explain how quantitative variables were handled in the analyses. If applicable, describe which groupings were chosen and why	5-6
Statistical methods	12	(*a*) Describe all statistical methods, including those used to control for confounding	6-7
		(*b*) Describe any methods used to examine subgroups and interactions	n/a
		(*c*) Explain how missing data were addressed	6
		(*d*) If applicable, explain how loss to follow-up was addressed	6-7
		(*e*) Describe any sensitivity analyses	n/a
**Results**			

For peer review only

Participants	13*	(a) Report numbers of individuals at each stage of study—eg numbers potentially eligible, examined for eligibility, confirmed eligible, included in the study, completing follow-up, and analysed	S1
		(b) Give reasons for non-participation at each stage	n/a, non-response to survey
		(c) Consider use of a flow diagram	S1
Descriptive data	14*	(a) Give characteristics of study participants (eg demographic, clinical, social) and information on exposures and potential confounders	Table 1 & 2
		(b) Indicate number of participants with missing data for each variable of interest	Table 2 footnote
		(c) Summarise follow-up time (eg, average and total amount)	5
Outcome data	15*	Report numbers of outcome events or summary measures over time	7-10
Main results	16	(*a*) Give unadjusted estimates and, if applicable, confounder-adjusted estimates and their precision (eg, 95% confidence interval). Make clear which confounders were adjusted for and why they were included	7-10
		(*b*) Report category boundaries when continuous variables were categorized	Table 1 & 2
		(*c*) If relevant, consider translating estimates of relative risk into absolute risk for a meaningful time period	n/a
Other analyses	17	Report other analyses done—eg analyses of subgroups and interactions, and sensitivity analyses	6-7
**Discussion**			
Key results	18	Summarise key results with reference to study objectives	11
**Limitations**			
Interpretation	20	Give a cautious overall interpretation of results considering objectives, limitations, multiplicity of analyses, results from similar studies, and other relevant evidence	11-12
Generalisability	21	Discuss the generalisability (external validity) of the study results	11-12
**Other information**			
Funding	22	Give the source of funding and the role of the funders for the present study and, if applicable, for the original study on which the present article is based	13

*Give information separately for cases and controls in case-control studies and, if applicable, for exposed and unexposed groups in cohort and cross-sectional studies.

Note: An Explanation and Elaboration article discusses each checklist item and gives methodological background and published examples of transparent reporting. The STROBE checklist is best used in conjunction with this article (freely available on the Web sites of PLoS Medicine at http://www.plosmedicine.org/, Annals of Internal Medicine at http://www.annals.org/, and Epidemiology at http://www.epidem.com/). Information on the STROBE Initiative is available at www.strobe-statement.org.

For peer review only
